# Clinical analysis of 13 children with primary hyperoxaluria type 1

**DOI:** 10.1007/s00240-021-01249-3

**Published:** 2021-03-15

**Authors:** Jin-ai Lin, Xin Liao, Wenlin Wu, Lixia Xiao, Longshan Liu, Jiang Qiu

**Affiliations:** 1 Department of Nephrology, Guangzhou Women and Children’s Medical Center, Guangzhou, China; 2 Department of Organ Transplantation, The First Affiliated Hospital of Sun Yat-Sen University, Guangzhou, China

**Keywords:** *AGXT* gene, Children, Nephrocalcinosis, Primary hyperoxaluria type I

## Abstract

A retrospective statistical analysis of primary hyperoxaluria type 1 (PH1) in children from June 2016 to May 2019 was carried out to discover its clinical and molecular biological characteristics. Patients were divided into two groups (infant and noninfant) according to clinic type. There were 13 pediatric patients (male:female = 6:7) with PH1 in the cohort from 11 families (four of which were biological siblings from two families), whose median age of symptom onset was 12 months and median confirmed diagnosis age was 14 months. Infant type (6 patients) was the most common type. The infant type mortality rate (100%) was higher than the noninfant (14.3%) (*p* = 0.029). The incidence of renal failure in infant patients was 67%, while the noninfant was 14.3%. 8 of 10 patients with nephrocalcinosis (NC) (76.92%, 10/13) were diagnosed by radiological imaging examinations, including X-ray (3 patients), CT (4 patients) and MRI (1 patient). NC was an independent risk factor for renal insufficiency [OR 3.33, 95% CI (0.7–1.2)], *p* < 0.05). Nine types of *AGXT* gene mutations were found; 1 type, c.190A > T, were first reported here. The most common *AGXT* gene mutation was c.679_680del, which occurred in exon 6 (5 patients). The infant type is the most common type of pediatric PH, with a relatively higher ratio of renal failure at symptom onset and poor prognosis. NC is an independent risk factor leading to renal failure, and radiological imaging examination is recommended for patients with abnormal ultrasound examination to identify NC. *AGXT* gene detection is important for the diagnosis and treatment of PH1 in children.

## Introduction

Primary hyperoxaluria (PH) is a metabolic disease [1]. It is a kind of kidney disease caused by gene mutation resulting in the loss of some enzymes in oxalate metabolism and an increased oxalate concentration in the urine. It can cause stones, kidney calcification and even kidney failure. PH is divided into 3 types. Type 1 is the most common type in the clinic, accounting for 80%. PH1 is an autosomal recessive genetic disease caused by alanine glyoxylate aminotransferase (AGT) deficiency in the liver-specific peroxisomal system. Due to the rarity of Ph1, delayed diagnosis and missed diagnosis of Ph1 is prominent. There have been some case reports and studies on PH1 gene mutations in China, but there is still a lack of complete clinical data and research on PH1 in children. PH1 is one of the important causes of nephrocalcinosis (NC) and/or kidney stones in childhood. As patients with PH1 continue to deteriorate, PH1 is the main cause of renal failure. It has become a hotspot in the clinic. We retrospectively reviewed the clinical data of 13 pediatric patients with PH1 and analyzed their clinical, imaging and molecular biological characteristics to provide reliable data for future clinical diagnosis and treatment of PH1.

## Objects and methods

### Subjects

This was a retrospective study approved by the Ethics Committee of the Guangzhou Women and Children's Medical Center (2019050401). All patients or their guardians were informed of the study and signed written informed consent. Thirteen pediatric patients diagnosed with PH1 from June 2016 to May 2019 in the Department of Nephrology, Guangzhou Women and Children’s Medical Center or Department of Organ Transplantation, the First Affiliated Hospital of Sun Yat-Sen University were included. Inclusion criteria: (1) children with hematuria, renal calcinosis, kidney stones, other clinical symptoms and imaging findings; (2) with or without unexplained renal insufficiency; (3) recurrent calcium deposition or multiple calculi in the transplanted kidney; and (4) *AGXT* gene detection showing positivity. Patients with (4) and at least one point of the others above were involved. Exclusion criteria: without informed consent from patients or their guardians.

## Methods

### Clinical data collection

The clinical data, including sex, age of symptom onset, age of diagnosis, family history, laboratory examination, imaging examination (ultrasound, X-ray, CT, MRI) and genetic mutation analysis data, were collected.

### Molecular biogenetic testing

All patients underwent *AGXT* gene detection by sequencing the exons in the *AGXT* gene in the proband, and Sanger sequencing was used to verify the pedigree.

### Optical microscopic examination

The pathological tissue of the kidney isolated from patients undergoing renal biopsy was fixed in 10% neutral buffered formalin overnight, embedded in paraffin, cut into 4 μm thick sections and stained with hematoxylin eosin or periodic acid Schiff. Sections were observed by optical microscopy.

### Definition

Hypercalciuria was defined as [2] the ratio of urinary calcium (mg) to creatinine (mg) 2–4 h after breakfast being higher than the standard (aged 0–6 months ≥ 0.8; 7–12 months ≥ 0.6; older than 2 years ≥ 0.2).

PH1 classification [3] is divided into different types: Infant type (early symptom onset renal calcification and renal failure), child and adolescent type (recurrent urolithiasis and continuous progress of renal failure), adult type (delayed symptom onset renal stone), post transplantation type (diagnosis after kidney transplantation), and family type (diagnosis with family history before symptoms).

### Statistical analysis

Statistical analysis was performed using SPSS IBM 19.0. Quantitative data with a normal distribution are described as the mean ± SD; otherwise, data are described as medians with the interquartile range (IQR). Quantitative data with a normal distribution were compared using an independent *t* test; otherwise, the Wilcoxon rank-sum test was used. Qualitative data are described as frequencies and percentages. Pearson chi square and Fisher exact tests were used to compare the qualitative data. The correlation between renal calcinosis and renal function was analyzed by unconditioned logistic regression, which was expressed by the OR value. A *p* value < 0.05 (two-sided) was considered significant.

## Results

### Demographics

In total, 13 children (male: female = 6:7) from 11 families were included. The median age at symptom onset was 12 months (IQR 3 to 63 months), and the median age at diagnosis was 14 months (IQR 4 to 136 months). The age at diagnosis was older than the age at symptom onset (Wilcoxon rank-sum test, *U* = 2.94, *p* = 0.03). Four patients were biological siblings from two family, among which the younger brother was the proband and was diagnosed with uremia, while his elder sister was diagnosed due to family verification and had normal renal function. Their diagnosis was delayed for five years and three years, respectively.

### Clinical manifestation

At symptom onset, 5 patients presented renal failure, 3 with kidney stones, 2 with anemia, 1 with gross hematuria, 1 with urinary tract infection and 1 with metabolic acidosis. At the time of diagnosis, 10 patients had advanced to end-stage renal failure (ESRF). According to the PH1 classification, infant type was the most common (6 patients), followed by child and adolescent type (4 patients). In addition, 1 patient belonged to the familial type, and 1 patient belonged to the post-transplant type. The clinical classification for 1 patient was unclear.

### Imaging examinations

**Fig. 1:**
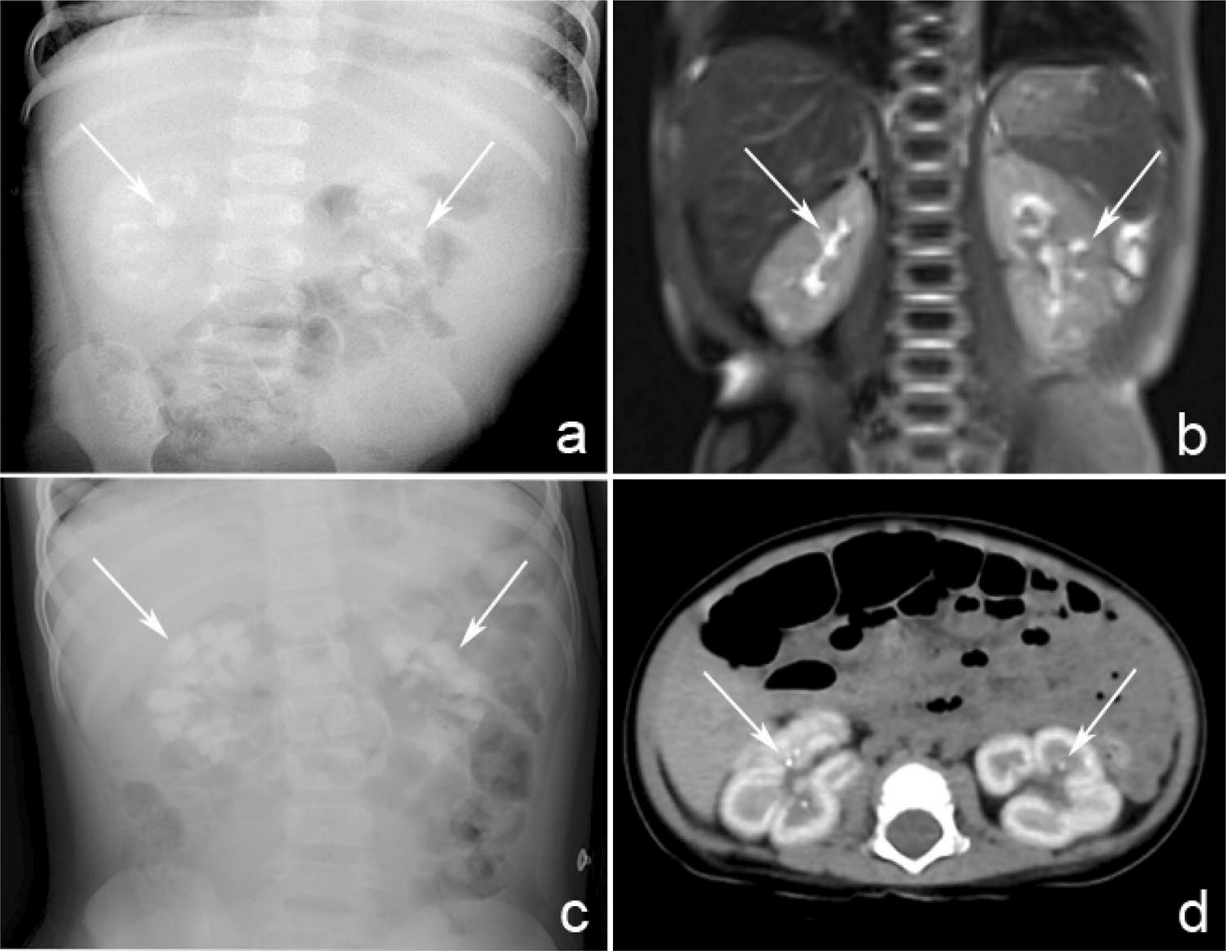
Radiological imaging examinations performed in patients with PH1. **a** Spot-like high-density imaging was found in bilateral kidneys on X-ray in patient II. **b** Signal of bilateral renal parenchyma increased diffusely on MRI T2WI in patient III. **c** The density of bilateral kidneys significantly increased on X-ray in patient IV. **d** CT scan in patient V showed under illumination of the contour bilateral renal contour, density of bilateral renal cortex significantly increased and multiple spotted high-density imaging in bilateral renal medulla (arrows)

**Fig. 2:**
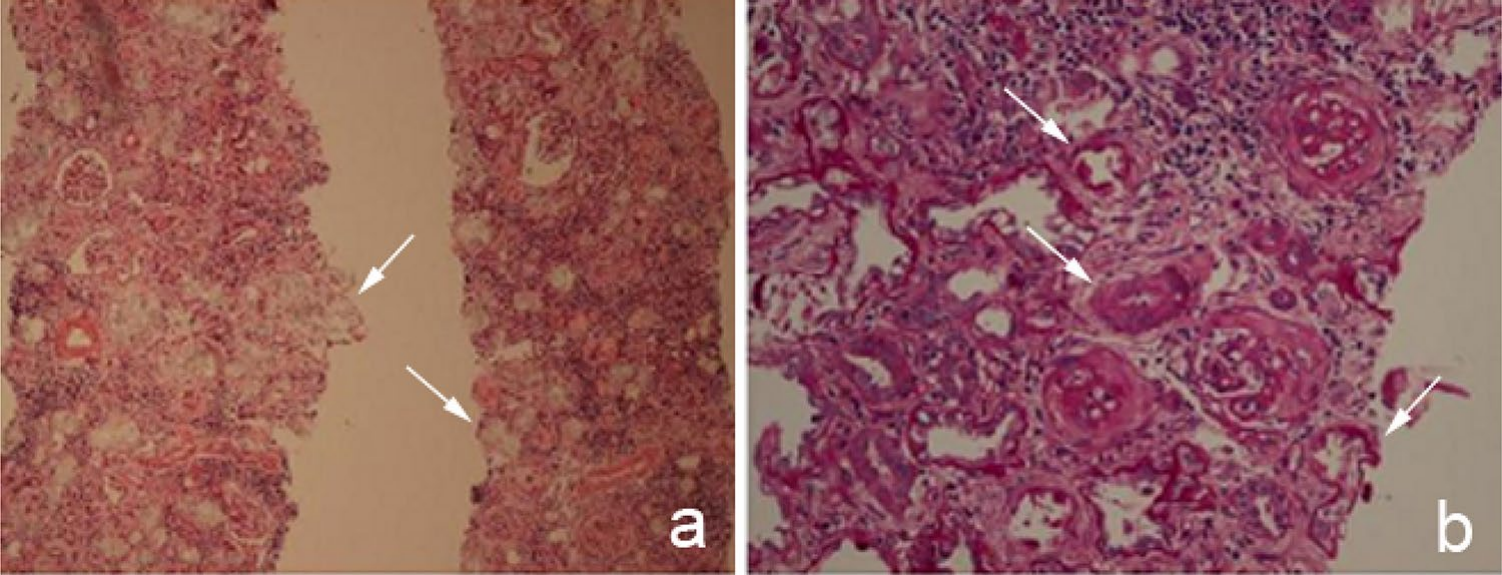
Renal histopathological examination in patient I. **a** Large amount of oxalate crystals in renal tubules for hematoxylin and eosin staining (white arrows, 10 ×). **b** Sclerosing glomerulus was observed for periodic acid Schiff staining (white arrows, 10 ×)

Imaging examinations included urinary ultrasound, abdominal X-ray, CT and MRI. Based on the examinations, 10 patients (76.92%, 10/13) were diagnosed with NC, among which 4 patients had multiple kidney stones, 2 had multiple stones in both kidneys, and 1 had small crystals in the medulla of both kidneys. Only two of these 10 patients were diagnosed with NC by ultrasound, and the others were diagnosed by radiological imaging examinations, including X-ray (3 patients), CT (4 patients) and MRI (1 patient), as shown in Fig. 1. Logistic regression analysis was carried out with NC and renal failure. The OR value was 3.33, the 95% CI was 0.66 to 3.45, and the P value was 0.004, suggesting that NC is an independent risk factor for renal insufficiency. One patient underwent renal biopsy, and optic microscopy findings showed that a large number of oxalate crystals were observed in the renal tubules, which was consistent with oxalate nephropathy with the majority glomerulosclerosis (Fig. 2).

### AGXT gene analysis

**Fig. 3:**
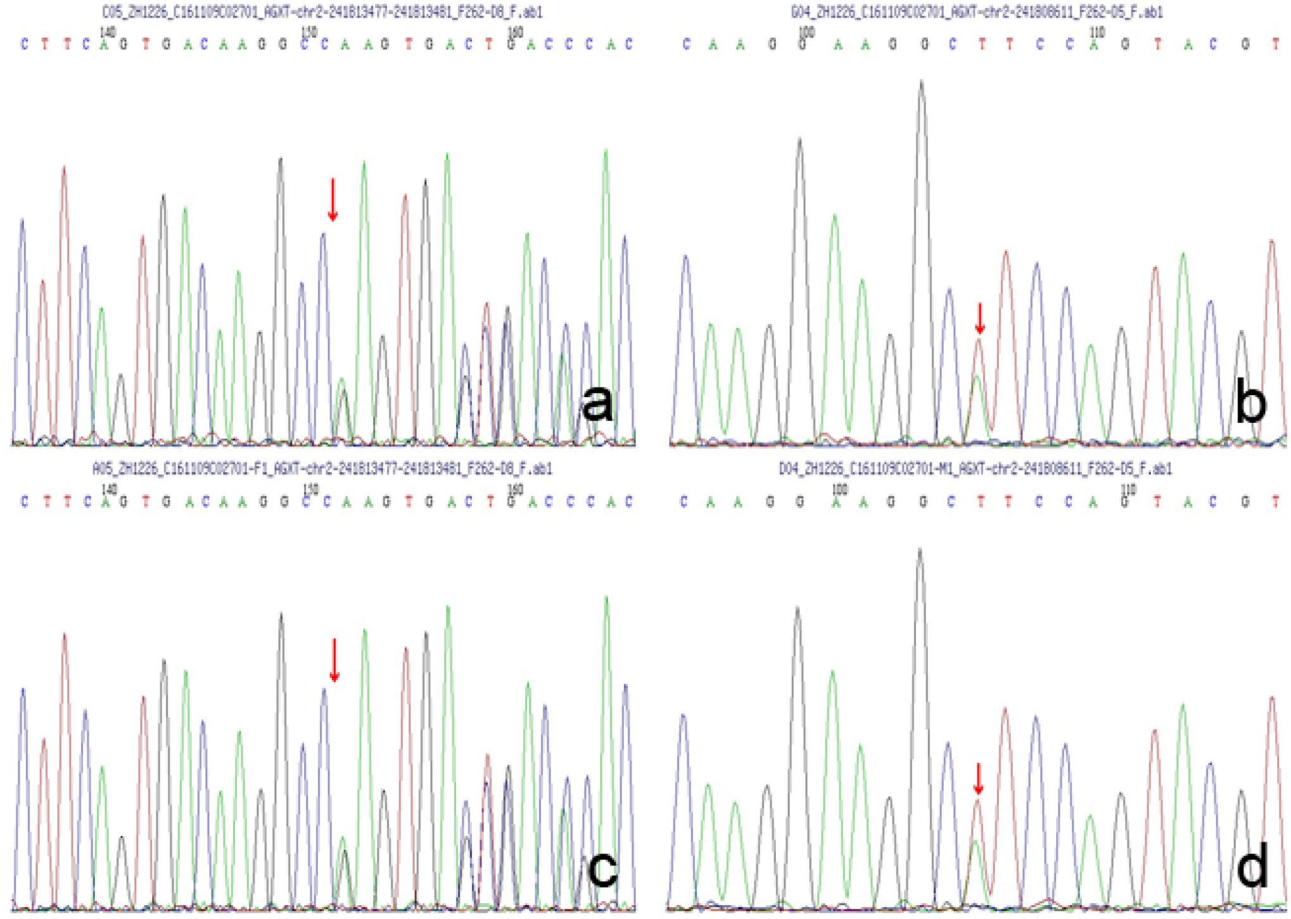
Peak map of *AGXT* gene sequencing in patient I with PH1. **a** *AGXT* gene mutation c.679_680 + 2delAAgt located in the sixth exon in the proband. **b** *AGXT* gene mutation *c.190A* > *T* (p.i64f) located in the second exon in the proband. **c** *AGXT* gene mutation *c.679_680* + *2delAAgt* located in the sixth exon in her father. **d** *AGXT* gene mutation *c.190A* > *T* (p.i64f) located in the second exon in her mother

For all patients, *AGXT* gene mutation was confirmed by second-generation sequencing and Sanger first-generation sequencing of target exons related to renal tubular disease. Five homozygous mutations and 8 complex heterozygous mutations in the *AGXT* gene were found. Nine types of *AGXT* gene mutations were found. We searched these gene mutation types in the HGMD database and ClinVar database on May 30, 2019, and the results showed that 8 types of *AGXT* gene mutations, including *c.1079G* > *A, c.2T* > *C, c.815_816insGA, c.215A* > *T, c.679_680* + *2delAAGT, c.32C* > *G, c.605T* > *A* and *c.679_680delAA,* had been reported. The remaining 1 type of *AGXT* gene mutation *c.190A* > *T* was first reported in our study. The most common *AGXT* gene mutation occurred in exon 6 (5 patients). In addition, four other types of *AGXT* gene mutations also occurred in exon 6. The pathogenicity of all mutations was predicted by SIFT, polyphen_2 and REVEL bioinformatics protein function prediction software. It is believed that these mutated genes are pathogenic, as shown in Fig. 3, except 190A > T.

### Treatment

Patients with renal failure, including one receiving combined liver and kidney transplantation and one single kidney transplantation, were treated with dialysis. Patients without renal failure treated with vitamin B6 had been following up (Table 1).

**Table 1:** Clinical data and laboratory examinations in 13 PH1 patients

Patient	Gender	Race	Age of onset (months)	Age at diagnosis (months)	Prognosis	Gene mutation	Mutation type	Urinary calcium/creatinine ratio	Serum creatinine (mmol/L)
1	Female	Asian	2	3	Died	c.679_680del, c.190A > T	Compound heterozygous	0.29	831
2	Female	Asian	12	14	Died	c.679_680del, c.190A > T	Compound heterozygous	0.15	862
3	Male	Asian	3	3	Died	c.1079G > A	Homozygous	0.33	592
4	Male	Asian	3	4	Died	c.2 T > C c.815_816insGA	Compound heterozygous	0.26	744
5	Male	Asian	59	116	ESRD(dialysis)	c.679_680delAA	Homozygous	0.15	1893
6	Female	Asian	122	178	Follow-up	c.679_680delAA	Homozygous	0.7	92
7	Female	Asian	5	8	Died	c.215A > T, c.679_680delAA	Compound heterozygous	0.2	370
8	Male	Asian	0.6	3	Follow-up	c.32C > G, c.605 T > A	Compound heterozygous	0.15	19
9	Male	Asian	110	148	ESRD(dialysis)	c.1079G > A	Homozygous	0.03	1287
10	Female	Asian	60	136	Post- transplantation( follow-up)	c.32C > G, c.815_816, insGA	Compound heterozygous	0.2	838
11	Female	Asian	132	132	Died for complication of transplantation	c.215A > T, c.1079G > A	Compound heterozygous	0.14	842
12	Female	Asian	5	8	Died	c.605 T > A, c.215A > T	Compound heterozygous	2.2	527
13	Male	Asian	63	156	Recurrence after transplantation	c.215A > T, c.815_816, insGA	Compound heterozygous	0.66	179

### Outcome

Seven patients died of complications or abandoned treatment with poor compliance, and six patients survived. Among these surviving patients, two patients received maintenance dialysis, while two had normal renal function. One patient treated with combined liver and kidney transplantation survived with normal renal function and has been followed up for 5 years. One patient was treated with kidney transplantation only, and he developed renal failure in the second year after kidney transplantation and was ultimately lost to follow-up. They were divided according to clinical type into the infant type group (6 patients) and the noninfant type group (7 patients). The ratio of renal failure to death was compared between these two groups (Table 2). Although the ratio of renal failure at symptom onset in the infant type group was 67%, which was higher than that in the noninfant type group (14.2%), there was no statistically significant difference between them (*p* = 0.086). The fatality rate in the infant type group was 100%, which was higher than that in the noninfant type group (14.2%) (*p* = 0.029).

**Table 2:** Comparison of the ratio of renal failure and death between the infant type group and the noninfant group

	Infant type	Non-infant type	χ^2^	*p*
Renal failure of initial symptom				
Yes	4	1	–	0.086^a^
No	2	6		
Death				
Yes	6	1	–	0.029^a^
No	0	6		

^a^Pearson χ^2^ and Fisher exact test

## Discussion

AGT is a pyridoxal-5-phosphate-dependent enzyme [1] that catalyzes the transamination of glyoxylate to glycine. AGT deficiency can lead to the accumulation of glyoxylates, which can convert to oxalate, leading to hyperoxalaemia or hyperoxaluria. Oxalate deposition in the urinary tract has become the main manifestation of PH1. PH1 is a rare disease with a prevalence of 0.7–2.9/1,000,000 in European populations [4]. The prevalence rate of PH1 is higher in Mediterranean countries where consanguineous marriage is permitted. Due to the lack of an effective registration system in China, data about the prevalence and incidence of PH1 in China are still absent.

In our study, the median age at symptom onset was 12 months, and approximately half of them were infant type and presented renal failure, which was consistent with other studies about pediatric PH1 in other countries [5–7]. We found that renal failure tended to be more common in infants than in noninfants, and the fatality rate in infants was several times higher than that in noninfants. These results suggested that patients with infant type PH1 present severe manifestations and poor prognoses. The data showed that the symptom onset of renal failure in the two groups was not statistically significant (*p* > 0.05), which may be related to the small sample size. In addition, this study found a highly positive correlation between hypercalciuria and the creatinine clearance rate, but it is still not clear whether hypercalciuria is an independent protective factor of renal function or an accompanying result when renal function changes, which still needs further study.

In our study, 10 patients (76.92%, 10/13) were diagnosed with NC; however, only two patients were identified by ultrasonic examination, and the others were identified by radioactive imaging examination. The sensitivity of ultrasound examination is not high. For patients with abnormally echogenic renal parenchyma or patients with unclear boundary structures of the cortex and medulla found by ultrasound examination, abdominal X-ray or non-enhanced CT examination is recommended for further examination [8]. In addition, we found that NC was an independent risk factor for renal insufficiency. Early detection of renal calcinosis and timely medical intervention can maintain residual renal function.

The coding gene of the AGT enzyme is *AGXT*, which is located at *2q37.3 *[9], with 11 exons in total. In addition, its coding protein contains 392 amino acids. A total of 237 pathogenic mutation sites in the *AGXT* gene have been reported in all exons, including point mutations, missense mutations, nonsense mutations and shear mutations. Twenty-five percent of these mutations were small deletions or insertions [10, 11]. 37% were *Gly170Arg,* while *c.33dupC*, *Phe152Ile*, *Gly156Arg* and *lle244Thr* were 11.0, 6.3, 4.5 and 2.7%, respectively. However, we found that the *c.679 delAA* (p.k228efs * 26) mutation located in exon 6 frequently appeared. Therefore, the gene mutation may be another specific high incidence hotspot in PH1 patients in China [12, 13], but a larger sample is needed for further study to confirm this hypothesis. The *c.190A* > *T* (p.i64f) mutation, located in the second exon, was first reported in our study and may be a newly mutated gene in PH1 patients. The pathogenicity of c.190A > T needs to be further verified by the studies of structure and function of the encoded protein. PH1 is a highly heterogeneous disease of clinical phenotype and genotype [14, 15]. In addition, no clear correlation between genotype and phenotype has been found thus far. For two patients who were biological siblings with the same gene mutation, the younger brother developed ESRD and needed maintenance dialysis treatment, while the renal function of his elder sister is still generally normal until now. Alfadhel et al. reported that the siblings had homozygous mutations of *c.187g* > *C* (p.gly63arg) in exon 2 of the *AGXT* gene, but the younger brother died of uremia at 4 months old, while his older brother still had no clinical manifestations at the age of 2 [16]. Therefore, biological differences such as the regulatory messenger microRNA associated with the transcription of the AGT enzyme leading to heterogeneity between genotype and phenotype may be a potential therapeutic target for PH1 in the future [17].

In our study, one patient treated with combined liver and kidney transplantation survived with normal renal function and has been followed up for 5 years. One patient was treated with kidney transplantation only, and he developed renal failure in the second year after kidney transplantation and was ultimately lost to follow-up. Combined liver and kidney transplantation was relatively effective compared with kidney or liver transplantation alone, which was consistent with other studies [18, 19]. At present, in addition to combined liver and kidney transplantation, there are many new treatment methods, such as neonatal liver cell transplantation, *AGXT* gene introduction and substrate reduction therapy, which are still in the research stage, and discovering more reliable and feasible treatment methods needs scientific progress.

To effectively reduce the missed diagnosis of pediatric PH1, quantitative testing of urinary oxalate or quantitative determination of oxalic acid urine excretion (urine oxalic acid/creatinine) should be used in patients with hematuria, renal calcinosis of unknown cause with or without hypercalciuria, and unexplained renal failure in early infant [21]. Molecular genetic testing should be used to confirm suspected cases. The quantitative determination of urinary oxalic acid is not available in our hospital, which is a limitation of our study.

## Conclusions

The infant type is the most common type in pediatric PH, with a relatively higher ratio of renal failure at symptom onset and poor prognosis. NC is an independent risk factor leading to renal failure, and radiated imaging examination is recommended for patients with abnormal ultrasound examination to identify NC. *AGXT* may have other mutations in addition to the reported mutations in PH1 patients.
